# Therapy Preferences in Melanoma Treatment - Willingness to Pay and Preference of Quality versus Length of Life of Patients, Physicians and Healthy Controls

**DOI:** 10.1371/journal.pone.0111237

**Published:** 2014-11-04

**Authors:** Ramona Krammer, Lucie Heinzerling

**Affiliations:** 1 Friedrich-Alexander-Universität Erlangen-Nürnberg (FAU), Erlangen, Germany; 2 Department of Dermatology, University Hospital Erlangen, Erlangen, Germany; University of Tennessee, United States of America

## Abstract

**Background:**

New melanoma therapies, like e.g. ipilimumab, improve survival. However, only a small subset of patients benefits while 60% encounter side effects. Furthermore, these marginal benefits come at a very high price of €110’000 per treatment. This study examines attitudes towards melanoma therapy options of physicians, healthy individuals and patients, their willingness to pay and preference of quality versus length of life.

**Methods:**

Based on findings from a focus group questionnaires were developed and pretested. After obtaining ethical approval and informed consent surveys were conducted in a total of 90 participants (n = 30 for each group). Statistical analyses were conducted using R.

**Findings:**

Attitudes vastly differed between healthy participants, physicians and melanoma patients. Whereas melanoma patients show a high willingness to endure side effects despite very small survival gains (down to 1 extra week) or even only hope with no survival benefit, healthy controls are more critical, while physicians are the most therapy adverse. Consequently, if given €100’000 and the free decision what to spend the money on the willingness to pay for therapy was much higher in the patient group (68%) compared to 28% of healthy controls and only 43% of the physicians, respectively. When lowering the amount of cash that could be received instead of ipilimumab to €50’000 or €10’000 to test price sensitivity 69% (+1%) and 76% (+8%) of melanoma patients, respectively, preferred ipilimumab over cash. When judging on societal spending even melanoma patients opted for spending on ipilimumab in only 21%.

**Conclusion:**

The judgment about the benefits of new treatment options largely differs between groups, physicians being the most critical against therapy. Price elasticity was low.

## Introduction

Recently, controversy has evolved concerning highly-priced drugs like ipilimumab with marginal benefit. Ipilimumab is approved for the treatment of metastatic melanoma, shows frequent side effects, a low response rate of 10–15% and a prolonged median overall survival by 2.1 months [1], [2] compared to standard chemotherapy dacarbazine (DTIC) and a higher percentage of survival beyond 2 years [3], [4] with some long-lasting responses [5]. Furthermore, these survival benefits come at unprecedented costs, i.e. with around €110’000 for the drug alone per treatment with ipilimumab as compared to €11’000 for a treatment cycle of DTIC. Moreover, ipilimumab frequently causes severe immune-related side effects including colitis, hepatitis, hypophysitis and various other side effects [6]. Another therapy option in patients with metastatic melanoma is palliative care which ensures maximum symptom relief and psychosocial support but mostly excludes tumor therapy. Palliative care can improve advanced cancer patients' quality of life and reduce pain by up to 71% [7]. According to a study a specialized inpatient palliative care with complex treatment for at least 21 days costs about €2’604 (reimbursement according to insurance companies payments; DRG version 2014).

The cost-benefit discussion among physicians is important since they have to decide over the value of each treatment for the individual patient considering side effects vs. response or prolongation of survival. Furthermore, there is also some financial responsibility for society that has to be taken into account. The cost burden of high-priced cancer drugs with marginal benefit is increasingly being criticized [8]. The drug cost only excluding laboratory costs or consultations for one melanoma patient benefiting from ipilimumab would be as high as €1.1 million, due to an estimated response rate of 10%. The cost of health care for cancer patients in the EU in 2009 amounted to €51 billion and contributes 40% of the total costs caused by cancer [9]. In general, costs for cancer care account for an increasing proportion of global spending on health care [10]. It is important to consider whether restrictions in the use of other health care technologies or additional health care funding will have to be introduced in order to pay for the new costly treatments [11].

Interestingly, individuals have different approaches in taking risks or chances. Consequently, depending on the individual a small chance of a long-term survival or an average survival prolongation of 2 months can be more appealing. The value of the therapy for the patient is difficult to assess. In the broader context the value of a therapy is characterized differently by different stakeholders [11]. The Pharmaceutical Research and Manufacturers Association (PhRMA) representing innovative biopharmaceutical research and discovery companies states that the value of medicine also depends on the reduction in other health care costs and better quality of life while the Pharmacy Benefits Advisory Committee, an independent expert body including health professionals/economists, doctors and consumer representatives that advises the Australian government on drug reimbursement considers affordability an important factor for the value of a drug [11]. The European Observatory on Health Systems and Policies, a governmental and WHO partnership, takes into account the product acceptability. According to these criteria drugs like ipilimumab would most probably not be highly valued. An important approach to assess these factors is including evaluations of costs and quality of life along with clinical endpoints, such as survival in clinical trials which is currently being done in the context of an ongoing clinical study. Nevertheless, physicians' willingness to initiate cancer therapy and spend additional health care resources is often, as in the case of ipilimumab, tied to an uncertain outcome [12].

Factors that influence a cancer patient's willingness to accept a therapy with a high probability of side effects in hope for a longer survival are not always tied to the outcome data or only treatment-related determinants such as the health benefit and toxicity, but also individual factors such as the patient's physical and mental condition [13]. Patients and physicians update their perceptions of the benefits and harms of a therapy as well as the level of uncertainty based on their own clinical experiences and individual assessment of treatments and their outcomes. Yet, patients' ability to understand probabilities of survival and side effects depend on their health numeracy skills, that are needed to understand medical numerical or statistical data and to use the information for decision-making [12]. This results in differences in the preferences of patients and physicians concerning different treatments [14].

This questionnaire-based study aimed to investigate the attitudes of physicians, healthy controls and melanoma patients towards ipilimumab, as an example for a high-priced drug with marginal benefit with regard to willingness to pay and acceptance of adverse events for a longer survival. We could show that patients prefer a longer survival regardless of side effects, response rates and price, in contrast to physicians who mostly do not accept side effects and a poor cost-benefit ratio.

## Materials and Methods

Hypotheses were generated within a focus group organized at the Department of Dermatology at the University Hospital of Erlangen. Participants included a melanoma patient, a relative of a patient, a palliative care specialist, an oncologist, a dermatologist, a medical ethics specialist, a surgeon experienced in care of oncological patients and two representatives of the Department of quality management, who deal with patient affairs. Participants were chosen to represent the different sectors involved in dealing with melanoma patients and to investigate different points of view. The participants were informed about the intention of our study, and invited in person and by email by the hosts (LH and RK).The questions included (i) whether the new drug should be applied as frequently as possible, (ii) which criteria were crucial for its recommendation, (iii) whether palliative medicine was an alternative, and (iv) whether higher costs were acceptable. With regard to prevention (v) prioritization of resources was discussed as a more cost effective means to decrease mortality. Participants were encouraged to bring forward their opinions and arguments for each topic. After the session, the statements were analyzed. The discussion was chaired by a private lecturer and senior dermatologist (LH) and documented by the first author (RK). Based on findings from the focus group and from literature three different questionnaires were designed for patients, healthy respondents and physicians and pretested. Pretests showed that patients had difficulties answering the life expectancy questions which led to adjustments for the final questionnaires. In total, 90 individuals participated in the survey with 30 respondents in each group group (File S1: Questionnaires for healthy participants, melanoma patients and health care providers).

### Ethics statement

Written ethical approval was obtained for both the focus group and the survey from the ethics commission of the medical faculty of the “Friedrich-Alexander-Universität Erlangen-Nürnberg”. Oral informed consent, has been obtained from the participants, as the data were analyzed and reported anonymously.

### Recruitment of the respondents

Outpatients and inpatients in the Department of Dermatology at the University Hospital Erlangen were asked to participate during the consultation. Patients who were considered unstable were excluded from the study in order not to confront them with difficult questions. Patients receiving their melanoma diagnosis for the first time or being informed about a worsening of their disease were also excluded. The sample of the healthy respondents was chosen to match age, gender and socioeconomic status of the patients. The questionnaires for the physicians were handed out in the Department of Palliative care, in the Department of Dermatology, in the Department of Hematooncology and to internal specialists and collected in drop-off boxes. In addition, two medical practices treating oncological patients participated in the study.

### Setting

Patients were assured that the answers would have no influence on their own therapy decision in any way and explained that the questions were merely hypothetical. Additionally, the interviewer was completely independent from the melanoma care team. The patients were explicitly told that their contribution would be anonymous and all data would be analyzed and published anonymously. Although the interviewer was present during the survey, the questionnaires could not be traced back to individual persons. Questions concerning the questionnaire were allowed. The patients were able and encouraged to talk about their feelings concerning the questionnaire at any time. They could also stop answering the questionnaire at any time if they felt uncomfortable or overstrained. All patients were seen by a physician independent of the study after completion of the questionnaire.

### Structure of the Questionnaires

In order to be able to compare the answers of patients and healthy respondents towards melanoma therapies, a common starting point was essential, meaning a determined state of health to which they were supposed to refer, when considering the questions. Thus, all respondents were confronted with a case example in which they should take the position of an advanced melanoma patient. The physicians were additionally asked to hypothetically treat the melanoma patient described in the case example.

The personal preferences for quality or quantity of life were assessed by asking to choose between two different therapy options with one allowing for a survival gain, but entailing worse side effects and the other a better quality of life. This was intended to be a trade-off between quality and length of life. The willingness to pay for a therapy that would possibly prolong life, but was likely to induce side effects, was examined by asking the respondents three times with decreasing sums if they would prefer ipilimumab or receiving the respective amount of money in cash. Moreover, the respondents had to choose on a five-point Likert scale from 0-4 (0 “I absolutely disagree”, 1 “I disagree”, 2 “I am undecided”, 3 “I agree”, 4 “I absolutely agree”) the most appropriate answer to statements that were partly inspired and adopted from the main arguments of a focus group discussion.

Parameters assessed within all three questionnaires included age, gender, children, family status, socioeconomic status and importance of belief (Table 1). Belief was measured on a four-point Likert scale from 0-3. In cancer patients we assessed if they had metastases or not. The physicians were grouped according to their status as a resident or consultant, their employment and their experience in cancer treatment measured in number of cancer patients per year and years of oncological practice.

**Table 1:** Characteristics of the study groups.

Characteristics	Study groups		
	Melanoma patients	Healthy respondents	Health care providers
	(n = 30)	(n = 30)	(n = 30)
Gender			
Male	67% (20)	50% (15)	47% (14)
Female	33% (10)	47% (14)	53% (16)
Age			
Range	25–87 years	19–90 years	28–52 years
(Median)	(57,5 years)	(52,5 years)	(37 years)
Family status			
Alone	7% (2)	20% (6)	20% (6)
With partner	53% (16)	40% (12)	40% (12)
With partner and child	27% (8)	27% (8)	37% (11)
Single parent	7% (2)	0% (0)	3% (1)
With others	3% (1)	10% (3)	0% (0)
Children			
Yes	80% (24)	60% (18)	37% (11)
No	17% (5)	33% (10)	63% (19)
Dependents			
Yes	33% (10)	13% (4)	NA^1^
None	57% (17)	80% (24)	NA
Belief			
None	13% (4)	27% (8)	33% (10)
Little	20% (6)	23% (7)	27% (8)
Medium	40% (12)	33% (10)	20% (6)
High	23% (7)	13% (4)	20% (6)
Education			
None	3% (1)	3% (1)	
Apprenticeship	43% (13)	40% (12)	
Master/technical college degree	20% (6)	27% (8)	
University degree	30% (9)	27% (8)	100% (30; for details see Table 2)
Employment			
Employee	43% (13)	37% (11)	87% (26)
Self-employed/	10% (3)	20% (6)	7% (2)
Other	40% (12)	40% (12)	
Net income per month			
Mean (**median**)	500–1000 Euros (**500–1000 Euros**)	3500–5000 Euros (**3500–5000 Euros**)	NA
Metastases			
Yes	53% (16)	NA	NA
No	40% (12)		

1 NA =  not assessed.

### Statistical analysis

For the statistical analysis of the questions the statistics program R version 2.15.2 was used. The correlations for ordinal data were calculated using the Spearman correlation coefficient using only complete cases. In this manner influencing factors on the given answers were examined. The associations including only ordinal data such as the questions with a Likert scale, age, income, work experience in years and number of patients were calculated using the linear by linear formula. For associations including non-ordinal data such as gender, family status, education, employment, medical department, the Chi^2^-test was used. The p-values in the figures were calculated using the Mann-Whitney-Wilcoxon-Test. If answers indicated a range of numbers, such as the years of experience with oncological patients or the number of oncological patients treated per year, the mean value was calculated.

The percentages indicated in the Results section exclude the missing data. Since the numbers were rounded it may occur that the sum of the numbers in the tables or figures may not exactly add up to 100%. If the Tables/Figures exclude “I absolutely agree” and “I absolutely disagree”, the percentage was summarized in the respective column “I agree” and “I disagree”.

## Results

Within this study a total of 90 respondents answered a survey tailored to melanoma patients, healthy respondents, and health care providers on valuation of a new treatment option, the anti-CTLA4-antibody ipilimumab, with marginal benefit, i.e. a low response rate, frequent side effects and a modest prolongation of median overall survival of 2 months. The effectiveness, side effects and costs of treatment with ipilimumab were illustrated and compared with standard treatment (chemotherapy with dacarbazine without effectiveness on overall survival), palliative care and prevention programs. Facts on the different treatment options were presented and respondents had to weigh life prolongation and quality of life and indicate their preferences for themselves and what they would recommend to others. In addition, the willingness to pay for different therapy or prevention options was assessed. Furthermore, attitudes and variables that influenced the respondents' preferences and decisions were evaluated. Of the distributed 100 questionnaires a total of 90 (90%) was recollected. 44 percent of respondents were female and the median age was 46 years. In Table 1 and 2 characteristics of respondents and health care providers are represented.

**Table 2:** Characteristics of the health care providers.

Characteristics	Health care providers (n = 30)
Specialty	
dermatology	37% (11)
oncology	23% (7)
palliative care	17% (5)
others	23% (7)
Education	
residents	40% (12)
senior physician	60% (18)
Oncological experience	
range	1–23 years
(mean)	(8 years)
(median)	(7 years)
Oncological patients treated per year	
range	0–1000 patients/year
(mean)	(211 patients/year)
(median)	(85 patients/year)

### General preferences on quality of life vs. length of life time

**Figure 1:**
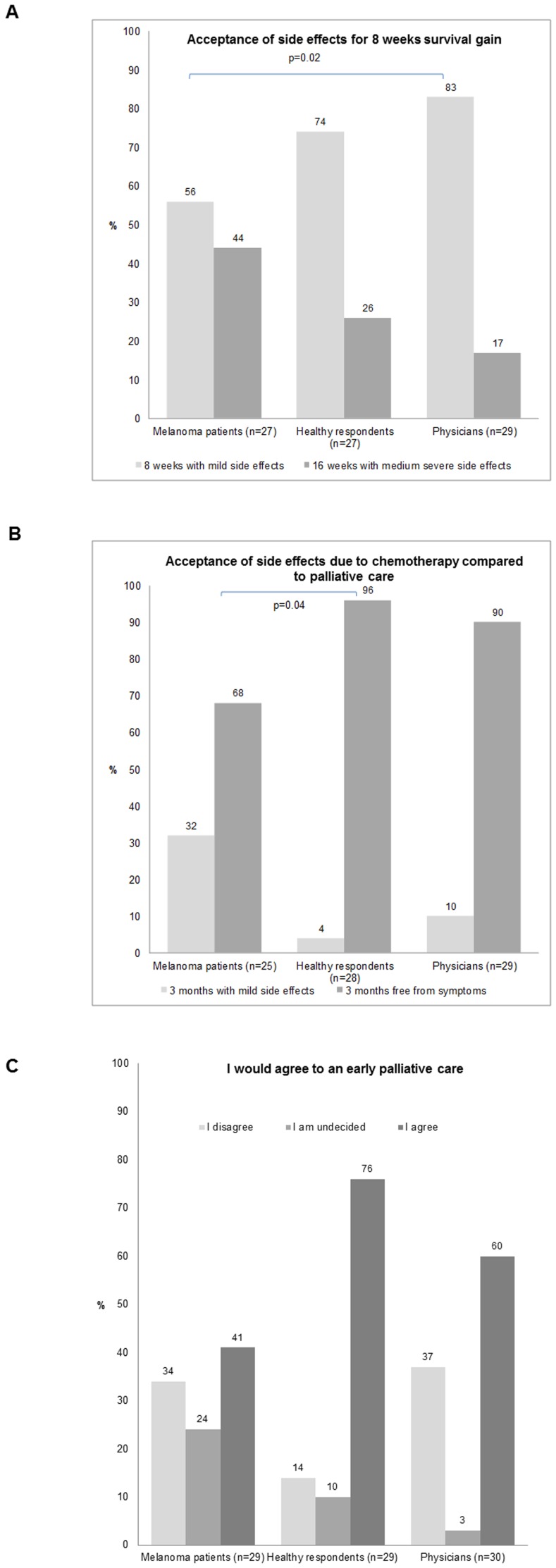
Acceptance of side effects. (A) Acceptance of side effects for 8 weeks survival gain. Preference of 16 weeks survival with moderate side effects with ipilimumab vs. 8 weeks survival with mild side effects with chemotherapy in melanoma patients, healthy respondents and physicians. Preference significantly differed between melanoma patients and physicians. **(B)** **Acceptance of side effects due to chemotherapy compared to palliative care.** Preference of 3 months survival with mild side effects with chemotherapy vs. 3 months survival free of symptoms with palliative care in melanoma patients, healthy respondents and physicians. Preference significantly differed with melanoma patients accepting tumor therapy with no survival prolongation more frequently than healthy respondents or physicians. **(C)** **Acceptance of early palliative care.** Acceptance of early palliative care in melanoma patients, healthy respondents and physicians. Numbers were rounded, it may occur that the sum of the numbers in the table may not exactly add up to 100%.

We hypothetically suggested people could pay for an end of life free of disease if they sacrificed on the length of lifetime (for specific questions view in supplemental materials questionnaire; question  =  q. 3). Overall, respondents were willing to renounce a mean of 11 years and a median of 3.5 years, if they could live in perfect health until the end of their life. Respondents commented however, that the renunciation on life years in exchange for a disease free end of life depended heavily on the severity and quality of symptoms you might otherwise suffer. Specifically, patients were prepared to renounce on a mean of 3 life years, while healthy people were prepared to renounce on 4 life years and physicians on 5 life years for perfect health until the end of life. The willingness to renounce on life years for health care providers was independent of the specialty of the health care providers (p-value = 0.52). Interestingly, this tendency was even more accentuated when this scenario became reality. Patients were the most prepared to accept side effects for a longer survival, whereas physicians were the most reluctant towards side effects with 44%, 26% and 17% of patients, healthy respondents and health care providers preferring moderate side effects and longer survival (16 weeks) to mild side effects and shorter survival (8 weeks; Figure 1A). These options were modeled to reflect real therapy characteristics with chemotherapy (dacarbazine) or ipilimumab, respectively (q. 4). When the survival gain was only one week these numbers were 19%, 19%, and 3% opting for longer survival in patients, healthy respondents and health care providers, respectively. When asked if they would prefer to live 4 months with medium severe side effects to 3 months free of symptoms in palliative care, one third of patients would rather live one month longer than have a higher quality of life at the end of their life. Still almost one quarter of the healthy group would choose the more intensive treatment for one month more whereas physicians were the most adverse towards the intensive therapy and favored palliative care in 90%. When asked about acceptance of a therapy no matter how severe the side effects might be if it would improve survival (q. 21) again patients were more prepared to take the risk (38%) as opposed to physicians with 3% and healthy respondents with 17%. Only 28% of the patients were reluctant to accept all possible side effects. The older the patients, the less likely they were willing to undergo treatment regardless of side effects (Spearman correlation coefficient  = -0.29; q. 21). Yet, we found that being parents was not associated with the acceptance of side effects for a longer survival (p-value = 0.34). More striking was the finding that even when no survival gain was offered by treatment still almost one third of melanoma patients were willing to undergo chemotherapy, live 3 months and suffer mild side effects of the tumor therapy instead of living 3 months free of symptoms in palliative care while 4% and 10% of healthy respondents and health care providers favored chemotherapy in that setting, respectively (Figure 1B).

Healthy respondents were more prepared to recommend ipilimumab than physicians. Physicians would not recommend ipilimumab in 87% compared to 44% of healthy respondents (q. 29). The physicians' recommendation was not associated with the specialty of the health care provider (p-value = 0.65). Patients were the most willing to take the risk of an intensive treatment for a mere 1% chance of survival gain, whereas the physicians were the most reluctant towards this option (Table 3, q. 13). The consent for therapy despite the low chance of a benefit was correlated with faith (Spearman correlation coefficient  = 0.10, q. 13), whereas the decision to recommend a therapy with 1% chance of a survival gain was not correlated with belief (Spearman correlation coefficient  = −0.05, q. 26; Table 4) and independent from the department of the health care provider (p-value = 0.49). The physicians' age was also not correlated with the willingness to accept ipilimumab for a mere 1% chance of a longer survival (Spearman correlation coefficient  = 0.00). Among patients age was inversely correlated with willingness to accept a 1% chance for a prolonged survival (Spearman correlation coefficient  = −0.11) i.e. older patients were less willing to take the risk. Likewise, older patients were the least willing to accept any side effects for a longer survival (Spearman correlation coefficient for age  = −0.29; Table 4). Furthermore, physicians' gender was not correlated with agreement to a therapy with 1% chance for a longer life (p-value = 0.96). Among patients there was neither an association between gender nor risk taking behavior and agreement with therapy (p-value = 0.63).

**Table 3:** Findings on survey questions regarding attitudes to treatment options of different study groups (melanoma patients, healthy individuals, physicians).

	I absolutely disagree	I disagree	I am undecided	I agree	I absolutely agree
“If a therapy (new drug) could prolong my life, I would always **agree** to it regardless of the side effects.” (q. 21)					
Melanoma patients (%)	10	17	34	21	17
Healthy respondents (%)	28	45	10	14	3
Physicians (%)	37	50	10	3	0
“I would **agree** to a hardly endurable treatment (ipilimumab) at any time, even if the probability of life prolongation was as little as one percent.” (q. 13)					
Melanoma patients (%)	21	31	24	14	10
Healthy respondents (%)	48	24	17	10	0
Physicians (%)	47	37	10	7	0
“I would **recommend** a hardly endurable treatment (ipilimumab) at any time, even if the probability of life prolongation was as little as one percent.” (q. 26)					
Healthy respondents (%)	25	36	18	18	4
Physicians (%)	63	30	3	3	0
“I would rather **recommend** ipilimumab than standard chemotherapy, since chemotherapy cannot prolong life.” (q. 35)					
Healthy respondents (%)	4	39	29	18	11
Physicians (%)	10	57	20	13	0
“I would **offer** ipilimumab rather to younger patients than to older ones.” (q. 39)					
Physicians (%)	7	7	10	63	13
“I would always apply ipilimumab if there is no contraindication.” (q. 28)					
Healthy respondents (%)	18	29	25	25	4
Physicians (%)	24	41	21	10	3
“If I had the money at my disposal and I could either pay ipilimumab or use it for other purposes, then I would rather spend the money in order to afford me something.” (q. 23)					
Melanoma patients (%)	10	14	34	31	10
Healthy respondents (%)	3	14	14	38	31
Physicians (%)	3	23	13	37	23

Numbers were rounded; it may occur that the sum of the numbers in the table may not exactly add up to 100%.

**Table 4:** Association of willingness to accept side effects for life prolongation (q. 21).

Willingness to accept side effects for life prolongation is associated with	Willingness to accept side effects for life prolongation is not associated with
**Age** (the older the more acceptance of side effects; Spearman correlation coefficient = 0.17, but among patients the older the patient the less the acceptance of side effects; Spearman correlation coefficient = −0.29)	**Employment** (p-value = 0.28)
**Belief** (the stronger the belief the more acceptance of side effects; Spearman correlation coefficient = 0.32, p-value = 0.00)	**Presence of metastases** (p-value = 0.18)
**Education** (the higher the education the less the acceptance of side effects; Spearman correlation coefficient = −0.27)	**Having children** (p-value = 0.34)

However, the decision on the tumor therapy with a 1% chance of a benefit healthy respondents and physicians made for themselves was different from their therapy recommendation to patients. Whereas 10% of the healthy people agreed with this intensive therapy for themselves (q. 13), they would recommend ipilimumab to patients in 21% (q. 26). In the case of the physicians it was the other way round. They were less likely to recommend this intensive therapy to patients than to accept it for themselves (3% vs. 7%; Table 3). Interestingly, 67% of physicians would not recommend ipilimumab over the standard chemotherapy dacarbazine despite ipilimumab's survival gain (Table 3, q. 35). The preference to treat with ipilimumab instead of dacarbazine was correlated with the number of cancer patients treated per year (Spearman correlation coefficient  = 0.18). However, the specific work experience with cancer patients expressed in years of practice was inversely correlated with preference of ipilimumab over chemotherapy, because of the potential survival gain (Spearman correlation coefficient  = −0.31, q. 35). Physicians were more likely (77%) to treat younger patients with ipilimumab (Table 3, q. 39) and this attitude was associated with belief (Spearman correlation coefficient  = −0.19; Table 5). Healthy respondents on the other hand were much more willing to prescribe ipilimumab than physicians with 29% agreeing to always apply ipilimumab if there was no contraindication compared to 14% of physicians (Table 3, q. 28).

**Table 5:** Influencing factors on willingness to preferably treat younger patients with ipilimumab (q. 39).

Willingness to preferably treat younger patients with ipilimumab is associated with	Willingness to preferably treat younger patients with ipilimumab is not associated with
**Belief** (the stronger the belief the less preferably younger patients treated; Spearman correlation coefficient = −0.19)	**Age** (Spearman correlation coefficient = −0.01)
	**Gender** (p-value = 0.17)

### Early palliative care

About one third of the patients rejected the idea of early palliative care, whereas the healthy respondents rejected it in only 14% (Figure 1C, q. 16). While 76% and 60% of healthy respondents and physicians, respectively, agree to early palliative care themselves, they would recommend it to patients in 83% and 80%, respectively (q. 36). Furthermore, there was a correlation between patients' preference for early palliative care and estimated life expectancy (Spearman correlation coefficient  = 0.21), i.e. the longer patients expected to live the more likely they were to accept early palliative care. Furthermore, believers were more prepared than non-believers to accept side effects for a longer survival. The physicians again were the most reluctant.

### Value of therapy

**Figure 2:**
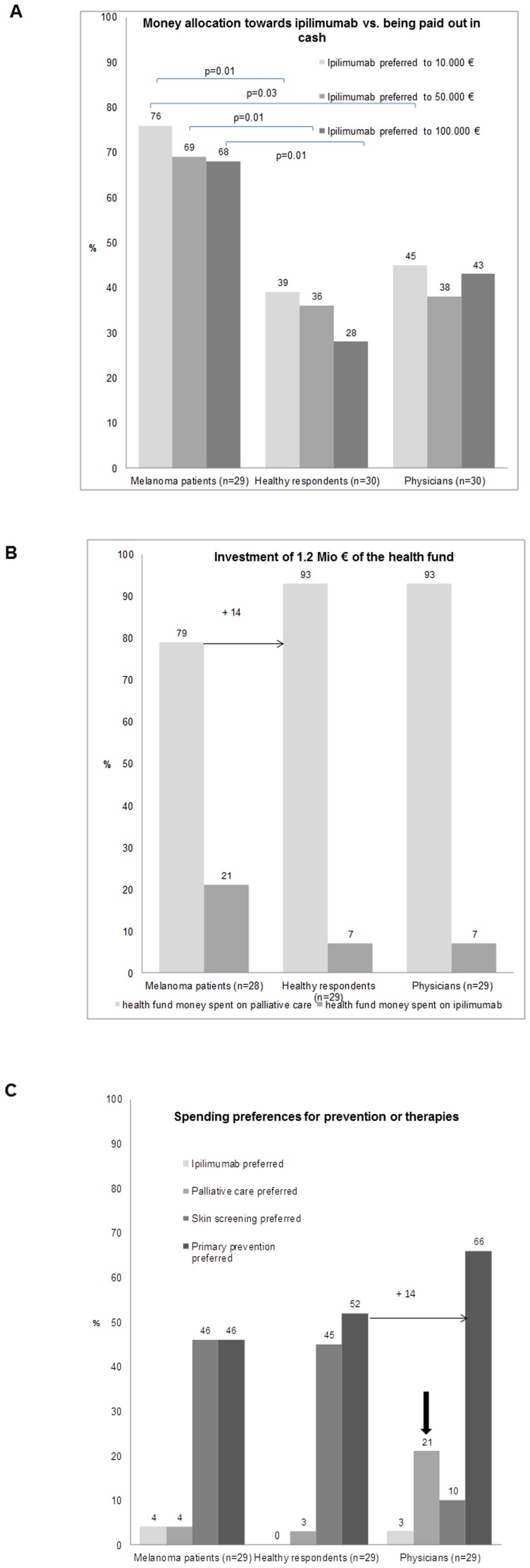
Valuation of different therapies and other health interventions. (A) Money allocation towards ipilimumab vs. being paid out in cash. Preference of allocation of €10.000, €50.000 and €100.000, respectively, towards ipilimumab vs. being paid out in cash, among melanoma patients, healthy respondents and physicians. Melanoma patients preferred therapy to cash significantly more frequently than healthy respondents or physicians, respectively. **(B)** **Investment of €1.2 Mio of the health fund.** Preference to invest €1.2 million for palliative care to help 245 melanoma patients vs. paying ipilimumab for 10 patients, with on average two months survival gain. **(C) Spending preferences for prevention or therapies.** Spending preference of €100.000 for ipilimumab, palliative care, skin screening or primary prevention in melanoma patients, healthy respondents and physicians. Physicians more frequently opt for spending on palliative care than other groups and prefer primary prevention efforts to screening.

To determine the value of ipilimumab with a two months survival prolongation but side effects, different amounts of cash were offered and choices had to be made between paying for the therapy and receiving the amount in cash (q. 10–12). Melanoma patients are by far the most willing to prefer ipilimumab over cash with 71% preferring therapy instead of money as opposed to 34% of healthy controls and 42% of physicians, respectively (Figure 2A). The higher the sum offered, the more healthy people decided to take the money in cash. In contrast, for the physicians the amount of money did not make a large difference. But about two thirds of the patients preferred the therapy instead of the money for any sum (€10’000, €50’000 and €100’000) opposed to only 28% of healthy respondents and 43% of physicians who preferred ipilimumab over €100’000. Interestingly, this higher preference of ipilimumab was the same when receiving the money (q. 10–12) or when asked to spend their own money (Table 3, q. 23). While 60% of physicians and 69% of healthy respondents wanted to use their own money for other purposes this was the case in only 41% of melanoma patients (q. 23).

### Societal spending preferences

Patients' decision making however differed when asked to spend money of the health fund for the sake of society as opposed to use the money for themselves. Confronted with social responsibility they chose in 79% to invest €1.2 million for palliative care and therefore help 245 melanoma patients, instead of paying ipilimumab for 10 patients, who would live on average two months longer (Figure 2B; q. 8). In this political context, in the other groups even fewer respondents favored ipilimumab (7%) while 93% chose to invest the money in palliative care. When allocating the money within society towards primary prevention, skin screening, treatment with ipilimumab or palliative care (q. 9) compared to the other groups physicians are the most willing to invest in palliative care (21%) and the least likely to spend money for screening (10%) whereas they clearly favor spending on primary prevention (66%; Figure 2C). In patients and healthy respondents there was an equal and high willingness to invest in screening and primary prevention, whereas the financing of ipilimumab or palliative care were mostly rejected with only 4% and 0% allocating money to ipilimumab and 4% and 3% allocating money to palliative care, respectively (Figure 2C).

Since the financial cost was a main concern for the prescription of ipilimumab, the respondents had to indicate if they were prepared to apply ipilimumab more often if it were cheaper. About one quarter of healthy respondents and physicians would apply ipilimumab more often if it were cheaper (Table 3, q. 33). For consultants the prescription was more affected if lowering the price than for residents (Spearman correlation coefficient  = 0.13).

**Figure 3:**
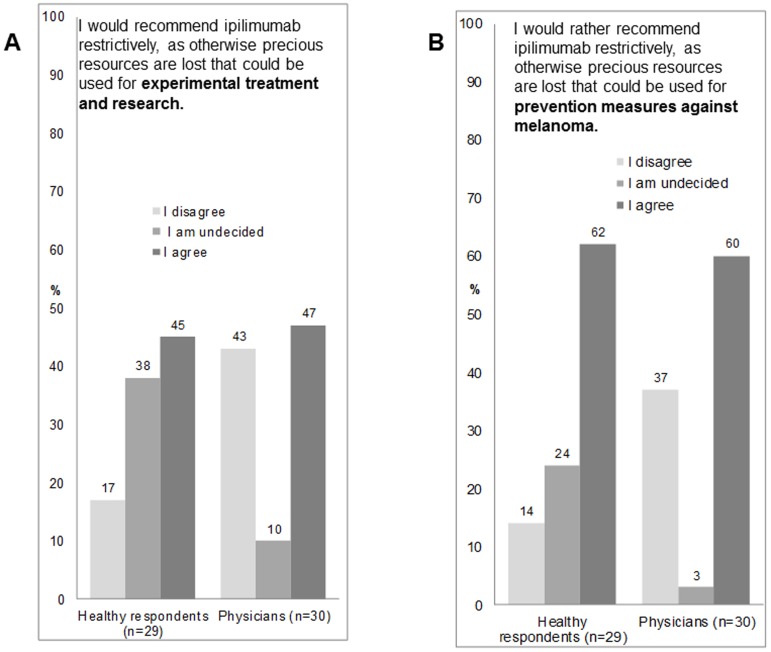
Restriction of ipilimumab for spending on other health interventions. (A) Restriction of ipilimumab to save money for research. Physicians are more frequently opposed to ration the drug than healthy respondents. (B) Restriction of ipilimumab to save money for prevention. Physicians are more frequently opposed to ration the drug than healthy respondents.

The willingness to prescribe ipilimumab if it were cheaper was inversely correlated with physician's years of experience in treating cancer patients (Spearman correlation coefficient  = −0.21), but not with the number of cancer patients treated per year (Spearman correlation coefficient  = −0.09). In view of the high cost burden that ipilimumab imposes, we asked if the prescription of ipilimumab should be restricted to save money for research or experimental therapies. While 43% of physicians were opposed to the restriction of ipilimumab this was only the case in 17% of healthy respondents. On the other hand 45% of the healthy respondents and 47% of the physicians were prepared to restrict use of ipilimumab and allocate the money to research (Figure 3A; q. 31). Healthy respondents and physicians are more prepared to restrict the use of ipilimumab for primary prevention than for research with 62% and 60% (Figure 3B; q. 32). The preference to save money for prevention programs or for research and experimental therapies instead of spending it on ipilimumab, was not associated with having children (p-value = 0.15; p-value = 0.73). There were no gender differences concerning the spending for prevention (p-value = 0.95) and research (p-value = 0.76). Consultants would rather save money for spending on research than residents (Spearman correlation coefficient  = 0.18). Furthermore, there was a negative correlation with the number of cancer patients treated per year (Spearman correlation coefficient  = −0.15) and the experience with cancer patients in years (Spearman correlation coefficient  = −0.12) i.e. the more experience the physician had in treating cancer patients the less likely they were to allocate the money to research.

Consultants would rather not limit spending on treatment in order to spend more for prevention (Spearman correlation coefficient  = −0.12). There was also a negative correlation with the number of cancer patients (Spearman correlation coefficient  = −0.17), and with the experience with cancer patients (Spearman correlation coefficient  = −0.09).

### Decision-making on the type of therapy

The influencing factors on the patient's decision were assessed. Two thirds of physicians (63%) and patients (66%), respectively, thought that as a patient their physician's advice was decisive in therapy decisions, while less than one third of healthy respondents (28%) agreed (q. 14). Half of the physicians (47%) and patients (48%) agreed that as a patient their family's advice on therapy decisions was decisive, while healthy respondents' opinions were split (38% agree and 38% disagree; q. 15). Most healthy respondents (41%) believed that physicians considered family advice as decisive in therapy decisions, while physicians disagreed (47%; q. 37). Physicians more frequently (77%) believed that their advice on therapy decisions was decisive than healthy respondents (45%; q. 38).

## Discussion

This is the first study on attitudes of melanoma patients, healthy respondents and physicians towards a new therapy option with marginal benefit, ipilimumab, in comparison to standard chemotherapy or palliative care. The purpose of this survey was to examine how the low response rates and frequent side effects as well as the costs of the new therapy are perceived by the different groups and how their perspectives differ. The valuation of therapies with marginal benefit is a difficult task in the individual setting as well as within society. This study confirmed that there are huge differences in therapy preferences of melanoma patients, healthy individuals and physicians. In general, the patient group strived for a longer life regardless of side effects and response rates, whereas physicians were reluctant towards side effects and therapy options with marginal benefit. Healthy respondents took a position between these extremes. Furthermore, the majority of patients preferred treatment as opposed to any amount of money in cash in spite of the merely marginal benefits. In contrast to that both, physicians and healthy respondents much more frequently preferred the cash than the costly treatment.

These findings could enrich the debate within society on therapies with marginal benefit as well as inform physicians on therapy preferences of patients which could enable better advice on the choice of therapy.

### Relevance of side effects

In our study older patients were less prepared than younger patients to undergo treatment regardless of side effects for a longer survival. Similarly, Stiggelbout et al. found that older patients preferred a better quality of life over means to live longer [14]. We found that having children was not associated with the preparedness to undergo therapy allowing for a longer survival regardless of side effects. However, Stiggelbout et al. had shown that Dutch cancer patients with children were more likely to pursue prolongation of life tolerating a lower quality of life [14]. These differences may be explained by the different study samples. Kuchuk et al. showed that breast cancer patients would risk a 38% chance of being dead to avoid having severe side effects like grade III/IV nausea/vomiting [15]. In our study fewer patients were reluctant to accept side effects (28%) while 38% of patients were prepared to accept them for a longer survival. These different findings may be explained by the fact that in our study these side effects were the price for a longer survival, and thus our patients' acceptance might be higher due to the promising survival gain. Yet, even for one week of additional life time about one fifth of melanoma patients and healthy respondents would accept the side effects of ipilimumab. Understandably, in Sun's study the most preferred health states included few or no side effects [16]. Nevertheless, our study shows that one third of patients and about a quarter of healthy people would rather live one month longer accepting multiple side effects than living 3 months free of symptoms supported by palliative care. In conclusion, it can be assumed that side effects do not matter as much as survival to patients. The will for survival seems to be stronger than for quality of life especially at the end of life. Similarly, Kuchuk et al. came to the conclusion that survival outweighed slowing cancer growth and maintaining quality of life across cancer stages [15]. We also tested how long the survival gain had to be to justify more severe side effects in return for a longer survival. For a survival gain of 8 weeks almost half of the patients accepted severe side effects, and for a one week survival gain still one fifth of the patients were prepared to do so. However, lung cancer patients who had not yet experienced severe symptoms were found to prefer a reduced risk of side effects over longer progression-free survival (PFS) [17]. A study in advanced ovarian cancer showed that if the therapy was equally effective, patients were willing to pay for avoiding side effects, as they chose a more expensive drug with a better adverse event profile accepting co-payments [18]. New complementary approaches in melanoma include adjuvant drugs like melatonin [19] or vitamin D [20] since it could play a role in melanoma progression [21], [22] and -as investigated in *in vitro* models- inhibitors of melanogenesis [23], [24].

### Choices in the face of low response rates

The choices made in situations of low response rates are difficult to make and to advise on. Risk-taking preferences could bias the advice physicians give. When asked about the willingness to undergo a therapy with side effects for a 1% chance of a longer survival, patients agreed in 24%, healthy controls in 10% and physicians in 7%, which shows that there were still a substantial number of patients who would take even this very small chance. Similarly, Slevin et al. had examined if patients and physicians would accept an aggressive therapy for 1% chance of cure. Here, 53% of the patients, 13.5% of cancer nurses, 12.5% of the general practitioners, 20% of the oncologists, and 19% of the control group agreed [25]. Thus, potentially people are more prepared to accept a therapy with side effects if there is a minimal chance for cure than only for a longer survival and health care providers are less prepared to take this chance than patients. This is in line with the findings from a study investigated whether patients would rather make a “safe bet” or “hopeful gambles” concerning life prolonging therapies in patients with melanoma, breast cancer or other solid tumors. Here two therapy options were offered, one promising a certain number of months additional survival, the other promising a chance of a much longer survival, but also the chance of no additional survival at all. Given these options 77% of the cancer patients preferred the “hopeful gambles” even if the average survival of the two therapy options was equal. Among the melanoma patients 71% would even reject the on average more favorable chance of living two years for sure for a 20% chance of at least four and a half year survival [26]. In contrast, Jenkins et al. found that if only two out of five patients benefit from a drug costing £4’000 a month only 15% of cancer patients were willing to pay for such an unsure treatment, whereas healthy comparators were willing to pay in 20% of cases [27].

In our study the acceptance of a therapy with 1% chance of a longer survival was correlated with belief. Similarly, positive religious coping was associated with higher rates of intensive care aspiring life-prolongation near death in advanced cancer patients [28]. Patients answered in a survey that if they were critically injured they believed that God could still heal them even though the physicians told them there was no cure [29]. Moreover, a belief in a higher power in cancer patients was associated with higher requirements of cardiopulmonary resuscitation, mechanical ventilation, and hospitalization [29]. Similarly, patients whose spiritual needs were not satisfied by the clinical team more frequently died in an intensive care unit as opposed to staying in a hospice compared to those patients whose requirements were fulfilled [30]. Thus, end-of-life costs tended to be higher for religious patients whose spiritual needs were not met [30].

### What is the price a patient would be willing to pay for a longer life?

When given the choice for money (€10’000-€100’000 in cash) vs. therapy patients preferred the therapy in 76% to 68% of cases depending on the amount.

The economically most responsible decision to minimize years lost due to melanoma in a population cannot be applied when deciding for or as an individual patient. Not surprisingly, when faced with the decision two thirds of the patients were willing to spend up to €100’000 for ipilimumab. In line with our findings, a large US study with 150 cancer patient showed that 25% of melanoma patients were willing to pay at least €32’865 ($45’000) for a gamble choosing a therapy with a chance of a longer survival, but possibly resulting in no additional survival at all [26]. Patients would even be willing to pay €39’703 ($54’362) for an uncertain chance of longer survival and €26’515 ($36’305) for an additional year of survival [26]. In a European study involving patients, care givers and the general public 40% of participants were prepared to pay even up to €200’000 or more for an additional year of life [31]. In the USA, only 24% of people were willing to pay €146’068 ($200’000) plus for an extra year of life [31]. Similarly, Jenkins et al. found that patients would be willing to pay for expensive anti-cancer drugs €4’860 (£4’000) per month from personal funds (20%), they would even re-mortgage their house (22%) or ask family and friends for money (9%) [27]. In contrast to our findings here, participants from the general public were even more willing to pay for the drug with 31% using personal funds, 30% re-mortgaging their house and 15% asking family and friends [27]. Another study had found that 51% of members of the public would be willing to pay for expensive drugs if they could prolong survival by 4–6 months [27]. Likewise, patients in our study preferred ipilimumab even if they could have used the cash for other purposes.

### Is the price of a therapy relevant to the physician?

In our study, 23% of the physicians would recommend ipilimumab more often if it were cheaper. This shows the relevance of cost for therapy recommendations among physicians. The consultants were even more cost-aware than the younger residents. The more cancer patients a physician treated per year the less prescription practices depend on the price. Interestingly, a survey of Massachusetts oncologists found that 88% of oncologists thought that therapy cost should not influence their treatment decisions. However, most oncologists thought that a survival of 2–4 additional months was justifying a hypothetical incremental treatment expense of €51’124 ($70’000) [32]. This can be calculated into a cost-effectiveness threshold of €219’102 ($300’000) per quality adjusted life year (QALY) for the hypothetical therapy in the study [32]. For bevacizumab, the same threshold was calculated which is higher than the commonly cited standard of €36’517 ($50’000) per QALY. Again 78% of oncologists stated that cancer patients should not be deprived by “effective” care due to cost issues [33]. Nevertheless, in our study the physicians were the most reluctant to spend money on ipilimumab for themselves and preferred to take up to €100’000 in cash instead of the chance for a two months longer survival.

### Does patient age influence therapy recommendation?

Most physicians would rather treat younger patients with ipilimumab than elder patients. Similarly, physicians were found to be reluctant to suggest chemotherapy to older breast cancer patients [34] since older patients more often have comorbidities, rendering them unfit for chemotherapy. In immunotherapy occurrence of side effects much less depends on age or comorbidities. However, in a study by Foster et al. physicians favored younger patients for intensive therapies even if all other characteristics and comorbidities of the cancer patient were identical [35]. In the study by Leonard et al. life expectancy of the patient determined the patient's suitability for a cytotoxic therapy in 90% of cases that [34]. This is in contrast to our findings where patients with a shorter life-expectancy were less prepared to sacrifice life time for quality of life.

### Societal responsibility

When given the responsibility for money of the health fund to invest it for the sake of society compared to use the money for themselves 79% of the patients and 93% of healthy respondents and physicians, respectively, decided to rather invest €1.2 million for palliative care providing care for 245 melanoma patients, instead of funding ipilimumab for 10 patients, with an average survival gain of 2 months. Faced with the spending options primary prevention, skin screening, treatment with ipilimumab or palliative care, patients, healthy respondents and physicians alike were unwilling to invest in ipilimumab with 4%, 0% and 3%, respectively. In contrast to our findings, in a study from Britain, where costly cancer drugs are not always covered by the health fund 49% of cancer patients and 36% of the general public thought that the National Health Service (NHS) should always pay for all new cancer drugs available [27]. A different study showed that about two thirds of patients and care-givers, and half of the public thought that in their country too little money has been spent on fighting cancer [31].

### Decision-making on the type of therapy

When asked about therapy decisions two thirds of physicians (63%) and patients (66%), yet only 28% of healthy respondents thought their physician's advice was decisive in therapy decisions, whereas a questionnaire-based study showed that 78% of respondents wanted patients and families to decide on therapy options and only 41% thought that these decisions should be made along with physicians [31]. Physicians in our study were reluctant to treat patients with ipilimumab instead of chemotherapy even if a potential survival gain was lost. However, consultants were more likely to prefer ipilimumab than residents. The more cancer patients a physician treated per year the more he was likely to prefer ipilimumab over DTIC, yet the longer the physician had treated cancer patients the more likely he was to stick to DTIC. Likewise, Schildmann et al. discovered that physicians were influenced by various nonmedical factors including clinical experience [36].

## Conclusions

This study shows that physicians have a considerably more reserved attitude towards therapies with marginal benefits than their patients or healthy respondents. Being aware of these different valuations of quality and length of life could lead to a better understanding of the patients' needs and thus improve educating patients about therapy options and advising them on therapy decisions. In the societal context all groups were more critical to spending on therapies with marginal benefit. Larger studies on this topic are needed to further inform the discussion.

## Supporting Information

File S1 **Study Questionnaire.**
(PDF)

## References

[1] Hodi FS, O'Day SJ, McDermott DF, Weber RW, Sosman JA, et al (2010) Improved survival with ipilimumab in patients with metastatic melanoma. N Engl J Med 363: 711–723 NEJMoa1003466 [pii]; 10.1056/NEJMoa1003466 [doi] 20525992 PMC3549297

[2] Tarhini A, Lo E, Minor DR (2010) Releasing the brake on the immune system: ipilimumab in melanoma and other tumors. Cancer Biother Radiopharm 25: 601–613 10.1089/cbr.2010.0865 [doi] 21204754 PMC3011989

[3] Robert C, Thomas L, Bondarenko I, O'Day S, Weber J, et al (2011) Ipilimumab plus dacarbazine for previously untreated metastatic melanoma. N Engl J Med 364: 2517–2526 10.1056/NEJMoa1104621 [doi] 21639810

[4] Farolfi A, Ridolfi L, Guidoboni M, Nicoletti SV, Piciucchi S, et al (2012) Ipilimumab in advanced melanoma: reports of long-lasting responses. Melanoma Res 22: 263–270 10.1097/CMR.0b013e328353e65c [doi] 22516968

[5] Prieto PA, Yang JC, Sherry RM, Hughes MS, Kammula US, et al (2012) CTLA-4 blockade with ipilimumab: long-term follow-up of 177 patients with metastatic melanoma. Clin Cancer Res 18: 2039–2047 1078-0432.CCR-11-1823 [pii]; 10.1158/1078-0432.CCR-11-1823 [doi] 22271879 PMC3319861

[6] Voskens CJ, Goldinger SM, Loquai C, Robert C, Kaehler KC, et al (2013) The price of tumor control: an analysis of rare side effects of anti-CTLA-4 therapy in metastatic melanoma from the ipilimumab network. PLoS One 8: e53745 10.1371/journal.pone.0053745 [doi];PONE-D-12-27215 [pii] 23341990 10.1371/journal.pone.0053745 PMC3544906

[7] Bisht M, Bist SS, Dhasmana DC, Saini S (2010) Quality of life as an outcome variable in the management of advanced cancer. Indian J Med Paediatr Oncol 31: 121–125 10.4103/0971-5851.76194 [doi];IJMPO-31-121 [pii] 21584216 PMC3089919

[8] Fojo T, Grady C (2009) How much is life worth: cetuximab, non-small cell lung cancer, and the $440 billion question. J Natl Cancer Inst 101: 1044–1048 djp177 [pii]; 10.1093/jnci/djp177 [doi] 19564563 PMC2724853

[9] Luengo-Fernandez R, Leal J, Gray A, Sullivan R (2013) Economic burden of cancer across the European Union: a population-based cost analysis. Lancet Oncol 14: 1165–1174 S1470-2045(13)70442-X [pii];10.1016/S1470-2045(13)70442-X [doi] 24131614 10.1016/S1470-2045(13)70442-X

[10] Bosanquet N, Sikora K (2004) The economics of cancer care in the UK. Lancet Oncol 5: 568–574 10.1016/S1470-2045(04)01569-4 [doi];S1470204504015694 [pii] 15337487

[11] Ramsey S, Schickedanz A (2010) How should we define value in cancer care? Oncologist 15 Suppl 1: 1–4 15/suppl_1/1 [pii]; 10.1634/theoncologist.2010-S1-1 [doi] 20237209

[12] Mullins CD, Montgomery R, Tunis S (2010) Uncertainty in assessing value of oncology treatments. Oncologist 15 Suppl 1: 58–64 15/suppl_1/58 [pii]; 10.1634/theoncologist.2010-S1-58 [doi] 20237219

[13] Hurley KE, Chapman PB (2005) Helping melanoma patients decide whether to choose adjuvant high-dose interferon-alpha2b. Oncologist 10: 739–742 10/9/739 [pii];10.1634/theoncologist.10-9-739 [doi] 16249355 10.1634/theoncologist.10-9-739

[14] Stiggelbout AM, de Haes JC, Kiebert GM, Kievit J, Leer JW (1996) Tradeoffs between quality and quantity of life: development of the QQ Questionnaire for Cancer Patient Attitudes. Med Decis Making 16: 184–192. 8778537 10.1177/0272989X9601600211

[15] Kuchuk I, Bouganim N, Beusterien K, Grinspan J, Vandermeer L, et al (2013) Preference weights for chemotherapy side effects from the perspective of women with breast cancer. Breast Cancer Res Treat 142: 101–107 10.1007/s10549-013-2727-3 [doi] 24129976

[16] Sun CC, Bodurka DC, Donato ML, Rubenstein EB, Borden CL, et al (2002) Patient preferences regarding side effects of chemotherapy for ovarian cancer: do they change over time? Gynecol Oncol 87: 118–128. S0090825802968071 [pii] 12468352 10.1006/gyno.2002.6807

[17] Bridges JF, Mohamed AF, Finnern HW, Woehl A, Hauber AB (2012) Patients' preferences for treatment outcomes for advanced non-small cell lung cancer: a conjoint analysis. Lung Cancer 77: 224–231 S0169-5002(12)00059-1 [pii]; 10.1016/j.lungcan.2012.01.016 [doi] 22369719

[18] Dranitsaris G, Elia-Pacitti J, Cottrell W (2004) Measuring treatment preferences and willingness to pay for docetaxel in advanced ovarian cancer. Pharmacoeconomics 22: 375–387. 2264 [pii]. 10.2165/00019053-200422060-00004 15099123

[19] Slominski A, Fischer TW, Zmijewski MA, Wortsman J, Semak I, et al (2005) On the role of melatonin in skin physiology and pathology. Endocrine 27(2): 137–48. 16217127 10.1385/ENDO:27:2:137 PMC1317110

[20] Slominski AT, Carlson JA (2014) Melanoma resistance: a bright future for academicians and a challenge for patient advocates. Mayo Clin Proc 89(4): 429–33 10.1016/j.mayocp.2014.02.009 24684870 PMC4050658

[21] Brożyna AA, Jóźwicki W, Janjetovic Z, Slominski AT (2013) Expression of the vitamin D-activating enzyme 1α-hydroxylase (CYP27B1) decreases during melanoma progression. Hum Pathol 44(3): 374–87 10.1016/j.humpath.2012.03.031. Epub 2012 Sep 17 22995334 PMC3529817

[22] Brożyna AA, Jozwicki W, Janjetovic Z, Slominski AT (2011) Expression of vitamin D receptor decreases during progression of pigmented skin lesions. Hum Pathol 42(5): 618–31 10.1016/j.humpath.2010.09.014. Epub 2011 Feb 2 21292298 PMC3078192

[23] Brozyna AA, VanMiddlesworth L, Slominski AT (2008) Inhibition of melanogenesis as a radiation sensitizer for melanoma therapy. Int J Cancer 123(6): 1448–56 10.1002/ijc.23664 18567001

[24] Slominski A, Zbytek B, Slominski R (2009) Inhibitors of melanogenesis increase toxicity of cyclophosphamide and lymphocytes against melanoma cells. Int J Cancer 124(6): 1470–7 10.1002/ijc.24005 19085934 PMC2628959

[25] Slevin ML, Stubbs L, Plant HJ, Wilson P, Gregory WM, et al (1990) Attitudes to chemotherapy: comparing views of patients with cancer with those of doctors, nurses, and general public. BMJ 300: 1458–1460. 2379006 10.1136/bmj.300.6737.1458 PMC1663147

[26] Lakdawalla DN, Romley JA, Sanchez Y, Maclean JR, Penrod JR, et al (2012) How cancer patients value hope and the implications for cost-effectiveness assessments of high-cost cancer therapies. Health Aff (Millwood) 31: 676–682 31/4/676 [pii];10.1377/hlthaff.2011.1300 [doi] 22492883 10.1377/hlthaff.2011.1300

[27] Jenkins VA, Trapala IS, Parlour L, Langridge CI, Fallowfield LJ (2011) The views of patients and the general public about expensive anti-cancer drugs in the NHS: a questionnaire-based study. JRSM Short Rep 2: 69 10.1258/shorts.2011.011050 [doi];SHORTS-11-050 [pii] 21969880 PMC3184012

[28] Maciejewski PK, Phelps AC, Kacel EL, Balboni TA, Balboni M, et al (2012) Religious coping and behavioral disengagement: opposing influences on advance care planning and receipt of intensive care near death. Psychooncology 21: 714–723 10.1002/pon.1967 [doi] 21449037 PMC3134563

[29] Phelps AC, Maciejewski PK, Nilsson M, Balboni TA, Wright AA, et al (2009) Religious coping and use of intensive life-prolonging care near death in patients with advanced cancer. JAMA 301: 1140–1147 301/11/1140 [pii]; 10.1001/jama.2009.341 [doi] 19293414 PMC2869298

[30] Balboni T, Balboni M, Paulk ME, Phelps A, Wright A, et al (2011) Support of cancer patients' spiritual needs and associations with medical care costs at the end of life. Cancer 117: 5383–5391 10.1002/cncr.26221 [doi] 21563177 PMC3177963

[31] Ramers-Verhoeven CW, Geipel GL, Howie M (2013) New insights into public perceptions of cancer. Ecancermedicalscience 7: 349 10.3332/ecancer.2013.349 [doi];can-7-349 [pii] 24044022 PMC3766630

[32] Schickedanz A (2010) Of value: A discussion of cost, communication, and evidence to improve cancer care. Oncologist 15 Suppl 1: 73–79 15/suppl_1/73 [pii]; 10.1634/theoncologist.2010-S1-73 [doi] 20237221

[33] Nadler E, Eckert B, Neumann PJ (2006) Do oncologists believe new cancer drugs offer good value? Oncologist 11: 90–95 11/2/90 [pii]; 10.1634/theoncologist.11-2-90 [doi] 16476830

[34] Leonard RC, Barrett-Lee PJ, Gosney MA, Willett AM, Reed MW, et al (2010) Effect of patient age on management decisions in breast cancer: consensus from a national consultation. Oncologist 15: 657–664 theoncologist.2009-0284 [pii]; 10.1634/theoncologist.2009-0284 [doi] 20551430 PMC3228002

[35] Foster JA, Salinas GD, Mansell D, Williamson JC, Casebeer LL (2010) How does older age influence oncologists' cancer management? Oncologist 15: 584–592 theoncologist.2009-0198 [pii]; 10.1634/theoncologist.2009-0198 [doi] 20495217 PMC3227998

[36] Schildmann J, Tan J, Salloch S, Vollmann J (2013) “Well, I think there is great variation…”: a qualitative study of oncologists' experiences and views regarding medical criteria and other factors relevant to treatment decisions in advanced cancer. Oncologist 18: 90–96 theoncologist.2012-0206 [pii]; 10.1634/theoncologist.2012-0206 [doi] 23287883 PMC3556262

