# Supplementary material for: Therapy Preferences in Melanoma Treatment - Willingness to Pay and Preference of Quality versus Length of Life of Patients, Physicians and Healthy Controls
Source: PLoS One. 2014 Nov 4;9(11):e111237. doi: 10.1371/journal.pone.0111237 (PMC4219712; doi:10.1371/journal.pone.0111237)
Supplement: File S1 — Study Questionnaire. (PDF) [file pone.0111237.s001.pdf]

Study Questionnaire

1.) Current state of health on a scale from 0% to 100%

Please indicate on the line below how you feel at the moment.

Worst imaginable medical condition (death)	0%	10%	20%	30%	40%	50%	60%	70%	80%	90%	100%	Best imaginable medical condition

----

Estimated life expectancy

2.) I think I am going to get ___________________________ years old.

Imagine there was a medication that made perfect health until the end of life possible. However, you would have to pay the treatment with lifetime and sacrifice some life years. Supposed you were going to get 80 years old, but had sanitary limitations such as daily pain in the back. You could be perfectly healthy until the end of your life with the medical preparation, if you renounced for example 5 life years and were only going to get 75 years old, but without any complaints

3.) I would be prepared to sacrifice _____________ life years, if I could live free of any sanitary complaints until the end of my life.

Case description

Please put yourself in the following situation

You have an advanced malign melanoma, which has already metastasized into inner organs. You suffer from pain in your bones, breathing difficulties and depression. You are educated that you suffer from an **uncurable** disease and that the average life expectancy in this stage of the disease rarely exceeded 9 months.

Therapy A – new drug

Your physician explains that there is a new drug that 10% of all eligible patients respond to, i.e. the tumor is getting smaller or disappears. On average patients live two months longer with this therapy. There is **no** chance for a cure. In more than half of all cases treatment-requiring adverse effects such as diarrhea, liver inflammation, hormone disorder, psychological symptoms, nausea, vomiting, severe abdominal pain and skin eruption occurred. The total therapy costs amount to about 120.000 Euro. The health insurance funds bear the costs.

Therapy B – standard chemotherapy

You are also educated about the standard therapy with chemotherapy that 6% of all eligible patients respond to, i.e. the tumor is getting smaller or disappears, whereby existing ailments could be alleviated. There is **no** chance for a cure and it is **not** prolonging life. Common side effects were loss of appetite, nausea, vomiting and alterations in the blood count. The price for a therapy cycle of three infusions of chemotherapy was about 10.500 Euro per therapy cycle. The health insurance funds bear the costs.

Therapy C – palliative care

Your doctor also offers you to enroll yourself into a palliative care program. Palliative care attends soothing pain and relieving discomfort as a consequence of the cancer in an inpatient or an ambulatory setting. Furthermore you receive psychological and clerical support at your request. You and your family will be advised and supported in medical, financial and social questions. By means of the cooperation of nurses, physicians, social workers and clerics a much more personal care than in a standard clinic is permitted. The costs lay within the range of 4.900 Euro on average per patient per year. The health insurance funds bear the costs.

Please decide for each pair of options which situation you would rather choose.

4.) ☐ Either two months of life with mild side effects with chemotherapy (Therapy B – chemotherapy)

☐ or four months of life with medium severe side effects. (Therapy A – new drug)

5.) ☐ Either eight weeks of life with mild side effects with chemotherapy (Therapy B – chemotherapy)

☐ or nine weeks of life with medium severe side effects. (Therapy A – new drug)

6.) ☐ Either four months of life with medium severe side effects (Therapy A – new drug)

☐ or three months free of complaints without any tumor therapy with social care at your request. This includes the possibility of visits by a cleric several times a week, financial consultancy by social workers, inclusion of the family in consultancy offers and a more personal care by nurses who have more time per patient available than on an ordinary department. (Therapy C – Palliative care)

7.) ☐ Either three months with mild side effects with chemotherapy (Therapy B – chemotherapy)

☐ or three months free of complaints without any tumor therapy with social care at your request. This includes the possibility of visits by a cleric several times a week, financial consultancy by social workers, inclusion of the family in consultancy offers and a more personal care by nurses who have more time per patient available than on an ordinary department (Therapy C – Palliative care)

The pharmaceutical industries have set prices for the presented therapy A (new drug) of about 120.000 Euros per treatment cycle. Since the financial resources of the health fund are limited, in daily life it has to be weighed in which case the new drugs should be applied.

Please indicate only one option in the following multiple choice questions.

8.) If you were authorized to decide upon the investment of 1.2 Mio Euro of our health fond, which decision would you take?

☐ A) I allow for the palliative care 245 patients by a specially trained team, whereby a better quality of life, but no life prolongation can be achieved.

☐ B) I invest in the new treatment and allow for a two months longer survival for 10 patients.

9.) If you would have to allocate 100.000 Euro from the health fond what would you do?

☐ A) I invest in one therapy cycle of the new treatment.

☐ B) I allow for the palliative care for twenty patients for one year.

☐ C) I allow for 7500 skin screening examinations, in order to detect about 28 malign melanomas early, when a cure is possible.

☐ D) I invest in prevention measures for the long-term decrease of melanoma incidence by 50% in 10 years.

10.) If you could choose between receiving 100.000 Euros in cash or therapy A, how would you decide?

☐ A) I would choose the new treatment option therapy A.

☐ B) I would renounce on any therapy and prefer the money in cash to fulfill myself one last wish or not to have any financial worries anymore.

11.) If you could choose between receiving 50.000 Euros in cash or therapy A, how would you decide?

☐ A) I would choose the new treatment option therapy A.

☐ B) I would renounce on any therapy and prefer the money in cash to fulfill myself one last wish or not to have any financial worries anymore.

12.) If you could choose between receiving 10.000 Euros in cash or therapy A, how would you decide?

☐ A) I would choose the new treatment option therapy A.

☐ B) I would renounce on any therapy and prefer the money in cash to fulfill myself one last wish or not to have any financial worries anymore.

Therapy decision

Please decide for each statement separately if you agree, even if the statements seem to be similar.

Please indicate only **one** option per statement.

**Patients perspective**	**I agree absolutely**	**I agree**	**I am undecided**	**I disagree**	**I disagree absolutely**
**13.)** I would agree to a hardly endurable treatment (therapy A) at any time, even if the probability of life prolongation was as little as one percent.	☐	☐	☐	☐	☐
**14.)** If I had to decide for a therapy with low response rates but frequent side effects (new drug), the advice of my attending physician would influence me the most.	☐	☐	☐	☐	☐
**15.)** If I had to decide for a therapy with low response rates but frequent side effects (new drug), the advice of my family and friends would influence me the most.	☐	☐	☐	☐	☐
**16.)** I would agree to an early palliative care (therapy C)./to palliative care alone. (physicians)	☐	☐	☐	☐	☐
**17.)** If I had suffered under chemotherapy (therapy B), I would not consent to another therapy (therapy A) with even more side effects but little chance for a longer survival.	☐	☐	☐	☐	☐

**<u>Patients perspective</u>**	**I agree absolutely**	**I agree**	**I am undecided**	**I disagree**	**I disagree absolutely**
**18.)** If my medical condition was better I would rather choose therapy A, as if my medical condition was worse.	☐	☐	☐	☐	☐
**19.)** If there was no chance for a cure, I would choose the offer of palliative care (therapy C).	☐	☐	☐	☐	☐
**20.)** I would rather make the most of my remaining life time (e.g. travel, visit family and friends) than undergo a stressing therapy (new drug) that has little chance of success and diminishes my quality of life.	☐	☐	☐	☐	☐
**21.)** If a therapy (new drug) could prolong my life, I would always agree to it regardless of the side effects.	☐	☐	☐	☐	☐
**22.)** If I had the money at my disposal and I could either pay the treatment or use it for other purposes, then I would try therapy A, even if it could be that I had only side effects and no benefit.	☐	☐	☐	☐	☐
**23.)** If I had the money at my disposal and I could either pay the treatment (therapy A) or use it for other purposes, then I would rather spend the money in order to afford me something (e.g. a cruise, invite family and friends).	☐	☐	☐	☐	☐

**Patients perspective**	**I agree absolutely**	**I agree**	**I am undecided**	**I disagree**	**I disagree absolutely**
**24.)** If I had the money at my disposal and I could either pay the treatment or use it for other purposes, then I would rather hand the money on (e.g. for the children).	☐	☐	☐	☐	☐
**25.)** If I felt bad, I would rather choose the offer of palliative care (therapy C).	☐	☐	☐	☐	☐

Physicians perspective

Please imagine you were in the position of a physician

Please read the following statements towards therapy decision as a physician and ***indicate only one option per statement.***

Physicians perspective	**I agree absolutely**	**I agree**	**I am undecided**	**I disagree**	**I disagree absolutely**
**26.)** I would recommend a hardly endurable treatment (therapy A) at any time, even if the probability of life prolongation was as little as one percent.	☐	☐	☐	☐	☐
**27.)** Since in the final stages of a cancer disease there is no curative therapy, I would try to emphasize the quality of life for the patient during treatment (therapy C).	☐	☐	☐	☐	☐
**28.)** I would always apply therapy A (new drug) if there is no contraindication.	☐	☐	☐	☐	☐
**29.)** I would generally recommend therapy A (new drug) to my patients.	☐	☐	☐	☐	☐
**30.)** I would not apply therapy A (new drug), since success can only be achieved for few patients and the costs and side effects are not worth it.	☐	☐	☐	☐	☐
**31.)** I would rather recommend therapy A (new drug) restrictively, as otherwise precious resources are lost that could be used for experimental treatment and research.	☐	☐	☐	☐	☐

**<u>Physicians perspective</u>**	**I agree absolutely**	**I agree**	**I am undecided**	**I disagree**	**I disagree absolutely**
**32.)** I would rather recommend therapy A (new drug) restrictively, as otherwise precious resources are lost that could be used for prevention measures against melanoma.	☐	☐	☐	☐	☐
**33.)** If therapy A (new drug) were cheaper, I would be more willing to take the risk and prescribe the therapy despite of its side effects more often.	☐	☐	☐	☐	☐
**34.)** I would rather apply therapy A (new drug) restrictively, since there are frequent side effects and only few patients have a benefit.	☐	☐	☐	☐	☐
**35.)** I would rather recommend therapy A (new drug) than standard chemotherapy (therapy B), since chemotherapy cannot prolong life.	☐	☐	☐	☐	☐
**36.)** I would rather recommend an early palliative care concept (therapy C), where the emphasis lies on quality on life.	☐	☐	☐	☐	☐
**37.)** In decisions for therapies with low response rates but frequent side effects (new drug), the advice of family and friends of the patient is decisive.	☐	☐	☐	☐	☐
**38.)** In decisions for therapies with low response rates but frequent side effects (new drug), the advice of the attending physician is decisive.	☐	☐	☐	☐	☐
**39.)** I would offer therapy A (new drug) rather to younger patients than to older ones.	☐	☐	☐	☐	☐

<u>Personal characteristics (Patients and healthy respondents)</u>

40.) Age ______ **41.)** Gender ☐ m ☐ f

42.) Family status:

☐ living alone ☐ living with partner ☐ living with partner and child ☐ single parent ☐ living with other persons

43.) Do you have children? ☐ Yes ☐ No

44.) Do you have someone who is depending on you (e.g. children, nursing case): ☐ Yes (who) ____ ☐ No

45.) How important is faith in your daily life?

☐ very ☐ medium ☐ little ☐ not at all

46.) Do you have a melanoma ☐ Yes ☐ No if yes: Do you have metastases? ☐ Yes ☐ No

<u>Sociodemographic characteristics</u>

47.) Please indicate which formation you have.

☐ none ☐ apprenticeship ☐ master/technical school degree ☐ university degree

48.) How is your employment?

☐ employed ☐ self-employed ☐ other (retirement)

49.) Please indicate in the column on the right in which range your monthly net income lies.

**Monthly net income**	Please indicate the appropriate range
Less than 500 Euro per month	
500 – 1.000 Euro per month	
1.000 – 2.000 Euro per month	
2.000 – 3.500 Euro per month	
3.500 – 5.000 Euro per month	
5.000 and more Euro per month	

Physicians

50.) Department ☐ Dermatology ☐ Oncology ☐ Palliative care ☐ Others

51.) Education status ☐ Assistent ☐ Specialist

52.) Employment ☐ employed ☐ self-employed

53.) How many oncological patients do you treat per year? ____________

54.) How many years have you been treating oncological patients already? ___________ years
